# Polysaccharide-Coated Magnetic Nanoparticles for Imaging and Gene Therapy

**DOI:** 10.1155/2015/959175

**Published:** 2015-05-19

**Authors:** Saji Uthaman, Sang Joon Lee, Kondareddy Cherukula, Chong-Su Cho, In-Kyu Park

**Affiliations:** ^1^Department of Biomedical Science and BK21 PLUS Centre for Creative Biomedical Scientists, Chonnam National University Medical School, Gwangju 501-746, Republic of Korea; ^2^Department of Agricultural Biotechnology and Research Institute for Agriculture and Life Sciences, Seoul National University, Seoul 151-921, Republic of Korea

## Abstract

Today, nanotechnology plays a vital role in biomedical applications, especially for the diagnosis and treatment of various diseases. Among the many different types of fabricated nanoparticles, magnetic metal oxide nanoparticles stand out as unique and useful tools for biomedical applications, because of their imaging characteristics and therapeutic properties such as drug and gene carriers. Polymer-coated magnetic particles are currently of particular interest to investigators in the fields of nanobiomedicine and fundamental biomaterials. Theranostic magnetic nanoparticles that are encapsulated or coated with polymers not only exhibit imaging properties in response to stimuli, but also can efficiently deliver various drugs and therapeutic genes. Even though a large number of polymer-coated magnetic nanoparticles have been fabricated over the last decade, most of these have only been used for imaging purposes. The focus of this review is on polysaccharide-coated magnetic nanoparticles used for imaging and gene delivery.

## 1. Introduction

Gene therapy is on the frontier of medicine. It involves preventing or treating serious diseases, including inherited disorders and some types of cancer and viral infections, by the use of genetic material. Several approaches are used, which can involve replacing a mutated gene that causes disease with a healthy one, inactivating a gene that is not functioning properly, or introducing a new gene to help treat a disease.

Successful gene delivery systems will depend on appropriate vehicles for carrying the therapeutic cargo (DNA or RNA) safely to the target cells [1] and the ability to target both tissues and cells with high specificity (to avoid toxicity to other cells) [2]. Moreover, the gene cargo must be released from encapsulating particles within specific intracellular compartments. Recent advances in nanotechnology and biotechnology have allowed the synthesis of novel nanoparticles that are more efficient gene carriers. Compared with viral gene delivery systems, these nanoparticles possess highly adjustable surface properties and vary in size and composition. A variety of nanovehicles have been synthesized using lipids, polymers, and inorganic materials. The polymer- and lipid-based nanovehicles are more suitable for delivering therapeutic genes, owing to their high reproducibility, ease of manufacture and functional modification, and safety (they are not carcinogenic) [3].

Currently, there are three techniques for delivering genes into suitable host tissues or host cells, including viral vectors, nonviral vectors, and electroporation. While viral vectors are highly efficient (80%–90%), they have a distinct drawback regarding the integration of nucleic acid sequences into the host genome, which may lead to unexpected outcomes such as inadequate expression of the gene or an immune reaction. The nonviral vectors that consist of cationic polysaccharides and lipids bind electrostatically with the cargo nucleic acid. Although the efficiency of transfection of host cells is only 20%–30%, nonviral vectors are safe and therefore may be preferable for clinical use because of good cell viability (80%–90%). Electroporation is the third method for introducing foreign genes into host cells. Although it can achieve a transfection rate of 50%–70%, more than half of the recipient cells do not survive the electronic stimulation.

**Figure 1:**
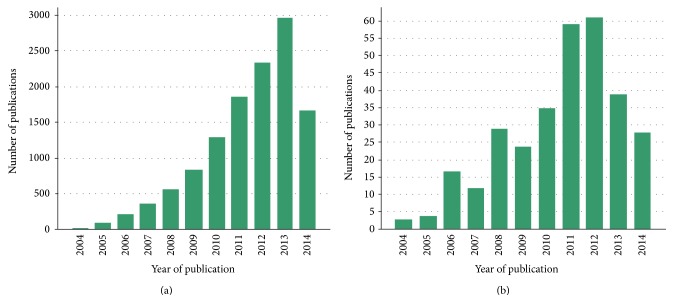
The number of scientific papers published over the last decade on (a) imaging and (b) gene delivery using MNPs. (Source: ISI Web of Knowledge: The Thompson Corporation. Search term: “magnetic nanoparticle imaging/gene delivery.” Date of search: August 2014).

Over the past two decades there have been a large number of investigations involving the use of magnetic nanoparticles (MNPs) for imaging and gene delivery applications (Figure 1). Research has centered on using MNPs in imaging modalities and as nanovectors for gene and drug delivery. These nanoparticles can act at the level of tissues or cells. Cellular internalization of nanoparticles beyond the cell membrane occurs either through endocytosis or phagocytosis. In some instances, nanoparticles can pass through the nuclear membrane (transfection applications). MNPs target tumors via passive or active methods. Passive targeting involves the extravasation of nanoparticles at the site of the tumor, where the microvasculature is leaky and hyperpermeable. Active targeting is based on the overexpression or exclusive expression of different receptors or epitopes on tumor cells [4].

Polysaccharides are a large family of polymeric carbohydrates comprised of long chains of monosaccharide units bound together by glycosidic linkages. Polysaccharides mainly consist of D-glucose, D-fructose, D-galactose, D-mannose, D-xylose, L-galactose, and L-arabinose [5]. They are very abundant in nature (biopolymer), low cost polymers and are easily modifiable. Apart from these, polysaccharides also have biological and chemical properties such as nontoxicity, biocompatibility, biodegradability, and high chemical reactivity [6]. In comparison with the non polysaccharide-coated MNPs, polysaccharide-coated MNPs have many advantages such as higher biocompatibility with the human body fluids; the elimination by white corpuscles from liver would be avoided and provides a steric barrier thereby preventing agglomeration [7]. Apart from all these the presence of a larger number of functional groups in the polysaccharide gives the polysaccharide-coated MNPs the capability of specificity in binding to the target site (tumor).

This review will mainly focus on the recent advances in the use of polysaccharide-coated MNPs for imaging and gene delivery. We discuss several strategies for tailoring polysaccharide-based MNPs to biomedical applications, with an emphasis on synthesis, surface modification, imaging, and gene delivery.

## 2. Synthesis of MNPs

There are several types of protocols for MNP synthesis. Some consist of a single step, while others involve multiple steps. However, all of these procedures have advantages and disadvantages; there is no universal solution for the preparation of magnetic particles. Most of these synthesis procedures use basic inorganic chemistry, especially iron chemistry, with the magnetic core material consisting of magnetite, iron-based metal oxides, or iron alloys. Some common methods are discussed in the following sections.

### 2.1. Precipitation

Precipitation methods for the preparation of magnetic particles are relatively simple chemical methods developed for the preparation of iron (II or III) ions in an aqueous solution. Basically, there are two types of precipitation methods: wet precipitation and coprecipitation. Wet precipitation is one of the oldest methods used to prepare magnetic particles [8]. Small particles are made by manipulating the pH of an iron salt solution. Iron oxide particles (Fe_3_O_4_) can be prepared using the coprecipitation method [9], where two stoichiometric solutions of Fe^2+^ and Fe^3+^ ions are mixed together, followed by the addition of a base. These methods also have drawbacks; the sizes of the resulting particles depend upon the pH, and the particles prepared by wet precipitation generally are larger. Because a large volume of water is needed for synthesis, the scale-up process is problematic. Moreover, for successful synthesis of Fe_3_O_4_, oxidation of the iron (II) precursor must be avoided as the oxidation leads to the transformation of magnetite (Fe_3_O_4_) to maghemite (Fe_2_O_3_) [10]. One of the most widely used methods for preventing the oxidation is via bubbling of N_2_ which also aid in reduction of the particle size [11]. Finally, it is virtually impossible to perform precipitation simultaneous with the addition of a protective coating to the magnetic particles, since maintenance of pH control is important and difficult.

### 2.2. Reverse Micelles

One of the classic examples of surfactant chemistry is the formation of micelles [12], which are generally formed when the concentration of amphiphilic surfactant molecules is such that appreciable numbers of micelles start forming (critical micelle concentration). While normal-phase micelles are formed in aqueous medium, reverse micelles are formed by surfactants in a nonpolar solvent such as hexane, in the presence of a very small amount of water. Iron oxide-based magnetic particles are prepared by slowly adding an inorganic precursor of iron (III) chloride, dissolved in aqueous medium with the subsequent addition to the oily medium, followed by the addition of pH regulators [13–17].

Magnetic particles prepared by a reverse micelle method are usually homogeneous in size, and inorganic coating material can also be added to the micelles during synthesis. Hence, with this method of synthesis, particle size can be controlled, and the particles can also be covered by an organic coating [18–23]. There are also disadvantages with this method. First, it is extremely difficult to coat the magnetic particles with organic compounds, because the monomers used in the coating remain in the organic phase of the micellar solution. Second, the size of the prepared particles is entirely dependent on the size of the micelles; it is not possible to prepare particles with sizes outside the range of sizes of the micelles, which is 20–500 nm [4, 24–28]. Third, it is extremely difficult to scale up the amount of final product, because of the large volume of organic solvents used during micelle preparation.

### 2.3. Thermal Decomposition

Recent advances in the fabrication of semiconductor and metallic nanocrystals have paved the way for synthesizing high-quality and monodispersed metal oxide particles, via the decomposition of organometallic complexes at high temperature [29]. Among iron oxide particles containing organometallic complexes, ferric and ferrous fatty acid complexes are widely used because of their low cost, low toxicity, and easily scaled-up synthesis for mass production [30–33]. Although this synthesis method is very popular in industry (because no solvents are needed for the synthesis), a drawback of this technique is the difficulty in controlling particle size of the nanoparticles.

### 2.4. Liquid Phase Reduction

The liquid phase reduction method is usually used for the reduction of magnetic or nonmagnetic metal oxides to magnetic metal (or metal alloys) by powerful reducing agents, such as NaBH_4_ and LiAlH_4_. NaBH_4_ is widely used as a reducing agent because it is soluble in both water and methanol [34–37]. Even though these hydrides are moisture sensitive and difficult to handle, the liquid phase reduction process has some advantages over other synthesis methods, because these hydrides are very active under mild conditions, and they can also penetrate the coating polymers.

## 3. MNPs for Imaging

MNPs have been applied to various biomedical fields, including imaging, where they have been used as contrast agents [38]. These nanoparticles consist of materials having magnetic properties, which include iron (Fe), gadolinium (Gd), cobalt (Co), and nickel (Ni). Since the early 1960s, iron oxides, as opposed to other metal oxides, have been used for magnetic separation. In 1978, Ohgushi et al. [39] first reported that iron oxides could shorten the T2 relaxation time (spin-spin relaxation time) of water and thereafter iron oxides have been extensively used as magnetic resonance imaging (MRI) contrast agents. Contrast agents used in MRI are divided into two categories; first one is paramagnetic compounds (including lanthanide like gadolinium) which have the property of reducing the longitudinal (T1) relaxation, thereby resulting in a brighter signal while the second group consists of super paramagnetic iron oxide which affects the transversal (T2) relaxation. An interesting property of super paramagnetic nanoparticles is that they become magnetized when an external magnetic field is applied and become quickly demagnetized upon removal of the magnetic field. This property confers a unique advantage for their use in biological systems, since after introduction into living systems; the particles are only active in the presence of an external magnetic field. Therefore, MNPs have long been extensively used as contrast-enhancing agents for MRI.

## 4. MNPs for Gene Delivery

Magnetic nanoparticles have been extensively used for a long time as contrast-enhancing agents for MRI [40] and are also being applied to gene therapy. To develop magnetic nanoparticles as effective gene carriers, the surface must be modified to allow electrostatic interaction between the surface and therapeutic molecule or must be conjugated to a cleavable linker. The applications of magnetic particles for gene delivery are being widely explored because of their unique characteristics.

### 4.1. Magnetofection-Based Gene Delivery

**Figure 2:**
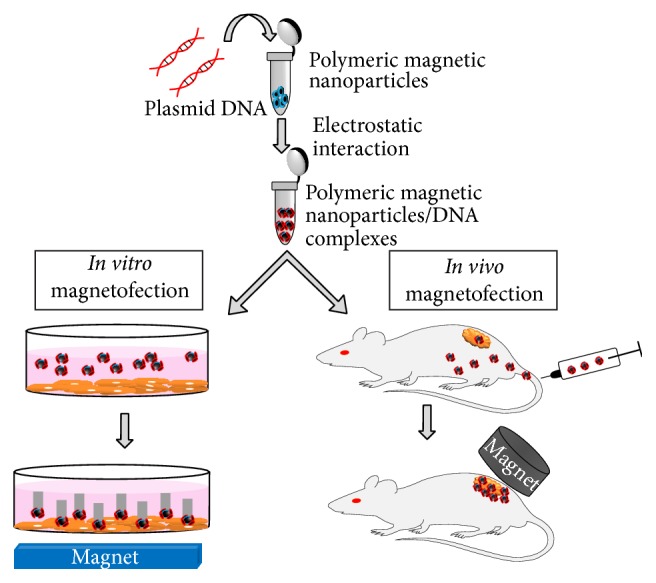
Schematic representation of* in vitro* and* in vivo* gene delivery using magnetofection (grey colour pattern on the left corner of the image represents the direction of movement of MNPs under the influence of magnet).

Magnetic nanoparticles are being used to increase transfection efficiency in a procedure called magnetofection [41–54], which employs a magnetic field to facilitate gene delivery, as shown in Figure 2. Advantages of the method include the rapid accumulation of the therapeutic agent at the target site, thereby reducing the transfection time and increasing the efficiency of transfection while decreasing cytotoxicity.

For* in vivo* magnetofection, the magnetic nanoparticles carrying therapeutic genes are generally injected intravenously. As the particles flow through the bloodstream, they remain at the target site because of the application of a very strong high-gradient external magnetic field. Once they are captured at the site, the magnetic particles carrying the therapeutic gene are taken up by the cells in the area, followed by the release of the gene via enzymatic cleavage of cross-linked molecules or degradation of the polymer matrix. If DNA is embedded inside or within the coating material, the magnetic field must be applied to heat the particles and release the gene from the magnetic carrier [55]. In this therapy MNPs were subjected to an oscillating magnetic field, which leads to the generation of heat via two mechanisms depending on the size of the particles: (a) Brownian mode: for MNPs < 100 nm in diameter, heat production is due to the friction between the oscillating particles, and (b) Neèl mode: for larger MNPs the heat is produced via the rotation of the magnetic moment with individual field oscillation [56].

The ideal polymers for surface modification of these nanoparticles should be biocompatible and nonimmunogenic and have high affinity for metal oxides. Once the nanoparticles are introduced to the physiological environment, they interact with hydrophobic surfaces, resulting in aggregation, opsonisation [57], and immediate clearance by the mononuclear phagocytic system (MPS). However, if hydrophobic particles are coated with hydrophilic polymers, interactions with plasma proteins can be prevented, resulting in increased* in vivo* circulation (due to reduced uptake by the MPS) [58]; coating allows nanoparticles to be multifunctional, allowing conjugation with drugs, genes, or imaging agents. Surface coatings of magnetic nanoparticles also prevent agglomeration and cytotoxicity and allow the addition of functional groups.

## 5. Polysaccharides as Surface Modifying Agents for MNPs 

**Figure 3:**
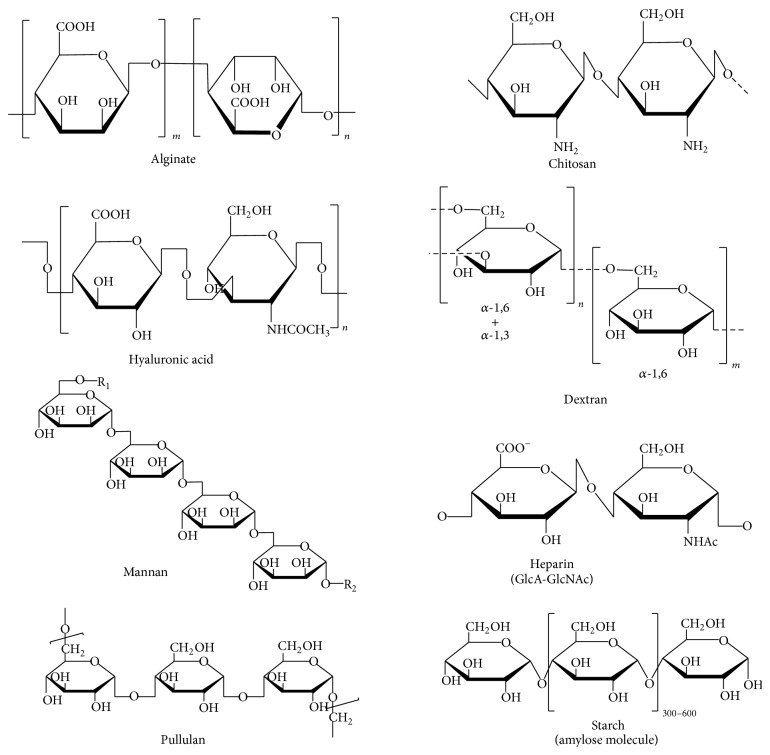
Structures of natural polysaccharides used for surface modification of MNPs.

The polysaccharides most commonly used for modifying the surface of MNPs include agarose, alginate, carrageenan, chitosan, dextran, heparin, pullulan, hyaluronic acid, and starch.  Figure 3 and Table 1 summarize the structures and properties of polysaccharides commonly used for coating MNPs. Most of the polysaccharide used for coating the MNPs are either negatively charged or neutrally charged (except chitosan which is positively charged); chemical modification was required for allowing the gene to interact with polymer coating on the MNPs.  Table 2 summarizes the various polysaccharides used for the coating of MNPs along with the different chemical modifications done to make them suitable for gene therapy.

### 5.1. Alginate

Alginates are anionic polysaccharides found in the cell walls of brown algae. They contain *β* (1-4) linked D-mannuronic acid and *α* (1-4) linked L-guluronic acid residues (Figure 3). Alginates have been widely studied because they form gels in the presence of divalent cations [59]. In contrast to gels with long-chain molecules that are held together by weak van der Waals forces, alginate gels are ionotropic. Alginates are widely used as biomaterials, especially as matrix supporting tissue repair and regeneration. They are biocompatible and nonimmunogenic and have chelating capabilities. Alginates are approved for use as polymers by the U.S Food and Drug administration (FDA) [60].

The standard synthesis method used for alginate-coated iron oxide nanoparticles consists of three steps. The first step involves gelation of alginate in a solution of ferrous ion, the second step involves the* in situ* precipitation of ferrous ion by alkaline treatment, and the third step involves the oxidation of ferrous hydroxide using oxidising agents such as O_2_ or H_2_O_2_ [80].

A two-step coprecipitation method has also been used for the preparation of alginate-coated magnetic iron oxide nanoparticles. This method involves the formation of the Fe_3_O_4_ particles through coprecipitation of ferric and ferrous ions by alkaline treatment and later the coating of the Fe_3_O_4_ particles by alginate [81]. The MNPs formed using this method were reported to have a core diameter of 5 to 10 nm, and after the MNPs were coated with alginate, the hydrodynamic diameter was found to be around 193.8 to 483.2 nm. When compared with the clinically used super paramagnetic iron oxide nanoparticles (SPIONs), the T2 relaxivity of the alginate-coated SPIONs was found to be higher; hence, alginate-coated SPIONs can be used as a negative MRI contrast agent.

Magnetized ferrofluid alginate microcapsules have also been prepared and were used to encapsulate the myoblast cell line C2C12. These ferrofluid alginate microcapsules were then implanted into the abdominal cavity of mice [61]. The cells showed similar viability as those encapsulated in unmodified alginate microcapsules. The ferrofluid alginate microcapsules remained intact and visible by MRI in both* in vitro* and* in vivo* experiments. The investigations of these magnetized ferrofluid alginate microcapsules demonstrated that they could be used for qualitative and quantitative MRI tracking of implanted capsules without invasive surgery. These properties could aid in the development of immunoisolation devices used in human gene therapy.

### 5.2. Chitosan

Chitosan is a biocompatible hydrophilic polysaccharide that is found in abundance in sea crustaceans and is appreciated for its biodegradable, nonimmunogenic, and nontoxic properties [82]. Chitosan is a copolymer of a 2-amino-2-deoxy-D-glucose and 2-acetamido-2-deoxy-D-glucose unit with *β* (1-4) linkages. It is obtained by the deacteylation of chitin. The chemical structure of chitosan is shown in Figure 3. The presence of functional amino and hydroxyl groups makes it a suitable candidate for the encapsulation of metal oxides, which result in hydrophilic, biocompatible, and stable particles. The cationic property of chitosan, mainly due to the presence of positively charged amino groups, allows it to interact with negatively charged nucleic acids. Furthermore, chitosan-coated MNPs can easily cross cell membranes and between chitosan-mediated opened tight junctions of epithelial cells [83, 84]. Moreover, the free hydroxyl and amino groups on chitosan allow the surface modification of SPIONS [69, 73, 85–91] by electrostatic interactions and physical adsorption, which anchor the polymer onto the surface of the iron and eliminate the need for a chemical cross linker. Chitosan and its dervatives have been the most widely used polysaccharides for fabricating magnectic nanoparticles for imaging and gene delivery (Tables 2 and 3).

A simple method for the preparation of chitosan-coated magnetic nanoparticles is the* in situ* precipitation of ferrous hydroxide using alkaline treatment in the presence of chitosan [92]. Another method used for synthesizing chitosan-coated MNPs includes the coprecipitation step followed by cross linking [93]. Polyethylene glycol- (PEG-) grafted chitosan was used to coat an iron oxide nanoparticle. The resulting nanoprobe could cross the blood brain barrier and target a brain tumor after the coating was conjugated with chlorotoxin, a tumor-targeting agent, and a near infrared fluorophore [62].

**Figure 4:**
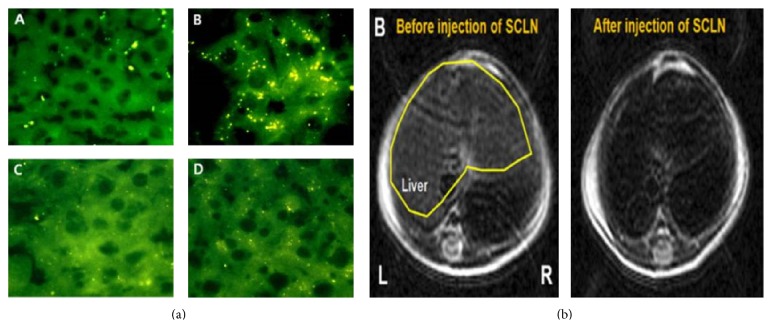
(a) Fluorescence image of mouse liver 48 h after intravenous injection of SPION-gene complexes and (b) nuclear image of ^99m^Tc-labeled SPIONs. Reprinted with permission from [79].

The delivery of genes to hepatocytes using SPIONs coated with chitosan and linoleic acid has been evaluated (Figure 4) [79]. The conjugates of chitosan and linoleic acid (a polyunsaturated fatty acid taken up by hepatocytes) were prepared via covalent linking between an amino group on chitosan and carboxyl group on linoleic acid. Coated 12 nm SPIONs were prepared via the self-assembly of the chitosan/linoleic acid conjugate. Coated SPION-gene complexes were formed by the ionic interaction between chitosan and negatively charged plasmid DNA coding for green fluorescent protein (GFP). After transfection of these complexes into hepatocytes, GFP expression was seen in hepatocyte cytoplasm. It was also reported that there was a significant increase in the GFP expression of* in vivo* hepatocytes of mice injected with these SPION-gene complexes.

### 5.3. Dextran

Dextran is another natural polysaccharide-based polymer used for various* in vivo* applications [94, 95]. Louis Pasteur first discovered it as a microbial product of wine. Dextran is a branched polysaccharide made up of glucose molecules with a linear backbone of repeating units of *α*-linked D-glucopyranosyl (Figure 3). Dextran-coated SPIONs have been commercially used as clinical contrast agents for MRI and have also shown promise in the nodal staging of cancers [96–98]. A coprecipitation method is most commonly used for the preparation of dextran-coated MNPs. The molecular weight of a dextran used for coating affects the stability, size, and morphology of MNPs and also affects coating efficiency. Low-molecular-weight dextran was found to result in a smaller particle of 77.8 nm, and increasing the molecular weight resulted in larger particle sizes ranging from 121.4 nm to 192.1 nm [99]. To increase the stability and functionality of dextran-coated magnetic particles, various functional groups such as a carboxymethyl group cross-linked to epichlorohydrin have been used to make cross-linked iron oxide particles (CLIOs), which are more stable than dextran-coated SPIONs of the same size [100].

Triple-labeled SPIONs coated with aminated dextran were investigated for their use as tags for hematopoietic CD34+ and neural progenitor cells [63]. The tripled-labeled SPIONs initially consisted of magnetic nanoparticles with a 5 nm monocrystalline core that was stabilised by cross-linked aminated dextran. The overall sizes of the coated nanoparticles were approximately 45 nm. The aminated cross-linked nanoparticles were then derivatized with a membrane translocation signal to make them target specific. The results suggested that dual-labeled, dextran-coated MNPs can be used for the efficient labeling of hematopoietic and neural progenitor cells, thereby allowing* in vitro* single-cell visualization by MRI. This technology may be useful for the advancement of stem-cell-based therapies.

In order to construct dextran-coated MNPs to carry genes, carboxymethyl dextran iron oxide nanoparticles (CDMN) were prepared by chemical coprecipitation [74]. The GFP gene was used as the reporter gene. The GFP-CDMN conjugate was prepared using the oxidation-reduction procedure. Magnetofection using the GFP-CDMN conjugate was evaluated for the human bladder cancer BIU 87 cell line. The results of this study demonstrated that application of an external magnetic field to a GFP-CDMN transfection resulted in higher transfection efficiency than transfection of conjugates without application of an external magnetic field.

In another study, MNPs containing Fe_3_O_4_-dextran-anti-*β*-human chorionic gonadotropin were prepared using the chemical coprecipitation method, and the feasibility of these MNPs as magnetofection agents was tested* in vitro* using various cell lines and* in vivo* using BalB/c nude mice [75]. First, dextran-coated MNPs were prepared by coprecipitation of dextran, ferric, and ferrous chloride under alkaline conditions. The dextran hydroxyl groups were oxidized to aldehyde groups using NaIO_4_. Anti-*β*-human chorionic gonadotropin monoclonal antibody was conjugated to the dextran-coated MNPs via the Schiff reaction. The resulting MNPs were less than 100 nm in size. The transfection efficiency of these nanoparticles was found to be significantly greater than the efficiency of liposomes (*P* < 0.05). The MNPs used in this study were also reported to combine specifically with the *β*-human chorionic gonadotropin in choriocarcinoma cells. It was also observed that application of a magnetic field and the use of immune targeting resulted in the concentration of MNPs in the targeted reticuloendothelial system and in the tumor. Even though the dextran-coated MNPs were initially approved by the EMA (European Medical Agency), it was later withdrawn owing to the agglomeration of the particles as a result of leaching of weakly bound dextran on the surface of the MNPs [101].

### 5.4. Hyaluronic Acid

Hyaluronic acid (HA) is an anionic nonsulphated polysaccharide widely distributed throughout connective, epithelial, and neural tissues. It is structurally composed of D-glucuronic acid and D-*N*-acetyl glucosamine, which is linked by alternating *β* 1-4 and *β* 1-3 glycosidic bonds (Figure 3). It is one of the major components of the tissue and extracellular matrix of vertebrates and interacts with the hyaluronan receptor, CD44 [64]. HA-CD44 binding contributes significantly to the intracellular signals that affect cell proliferation, differentiation, and migration. Because overexpression of CD44 is a hallmark of cancer angiogenesis and other types of tumor progression, HA is also used widely as a ligand in the fabrication of various nanotherapeutics targeted to cancer cells [102]. Thus, the surface modification of MNPs with HA is a very promising technique that can be used in the development of MNPs that target tumor cells or that can be used for imaging tumors. It has also been reported that production of hyaluronidase, which rapidly cleaves the glycosidic linkage of HA, is increased in various tumor cells compared to normal cells; therefore, HA-coated MNPs are not only biocompatible but have tumor-specific targeting properties [103, 104].

In order to develop HA-coated MNPs for targeted cancer imaging, HA was functionalized with dopamine for stable immobilization onto MNPs [64]. The MNPs used in this study were prepared by the thermal decomposition of iron oleate complex. For the surface immobilization of HA, the MNPs were transferred to an aqueous phase using cetyltrimethylammonium bromide (CTAB), which provided a cationic charge that led to the electrostatic interaction with negatively charged HA. For instantaneous surface coating, the HA-dopamine conjugates were prepared via EDC/HOBT chemistry. To evaluate the targeting effectiveness of HA-coated MNPs, T2-weighted gradient-echo MRI was performed using the CD44-positive cell line, HCT116, and a CD44-negative cell line, NIH3T3. The results of this study confirmed the targeting capability of the HA-coated MNPs via the CD44-HA receptor-ligand mechanism.

HA-coated MNPs have also been used for magnetofection of dendritic cells (DC) with DNA, which could lead to improved DNA vaccine delivery systems [77]. In this study, the magnetic vectors were comprised of polyethylenimine- (PEI) coated MNPs conjugated with HAs of different molecular weights to reduce cytotoxicity and to facilitate the CD44-mediated endocytosis of the particles into DC. To form quaternary polyplexes, the DNA was first mixed with HA to condense the DNA, and the mixture was allowed to interact with the positively charged PEI. This study demonstrated that the transfection efficiency of the magnetic polyplexes was significantly higher under the influence of the magnetic field at a low DNA dose and there was significant upregulation in the expression of CD86, indicating that the MNPs were taken up by HA receptors different from the CD44 receptors. Therefore, we can conclude that DC uptake of HA-coated MNPs is not CD44 dependent and that other HA receptors such as TLR2, TLR4, and CD38 might be involved.

### 5.5. Heparin

Heparin sulphate is a component of the extracellular matrix and is one of the members of the glycosaminoglycan family that can covalently attach to proteins to form proteoglycans. The most common unit making up heparan sulfate is glucuronic acid (GlcA) linked to* N*-acetyl glucosamine (GlcNac) (Figure 3). It has the highest negative charge density among the biological polysaccharides. Heparin is widely used as an anticoagulant medication. It has also been widely used in drug delivery and tissue engineering applications [105]. The fabrication of heparin-coated MNPs is generally by alkaline coprecipitation [106]; however, they have also been fabricated through the* in situ* coating [107]. Heparin coating increases the efficiency of MNP uptake because it increases the hydrophilic properties of MNPs, thereby facilitating the attachment of cells to the surface of the nanoparticle.

Glycol chitosan/heparin-immobilised MNPs have been fabricated as an MRI agent with tumor-targeting capabilities [65]. To induce ionic interaction between the iron oxide particles and glycol chitosan, gold was deposited over the iron oxide particles. These nanoparticles were stabilized by the ionic interaction between anionic heparin and cationic glycol chitosan. One of the most important advantages of using heparin in this study was the interaction between fibrinogen-derived products in the tumor and heparin in the MNPs. The interaction between fibrinogen products in the tumor and heparin in the MNPs suggests that heparin-coated MNPs have tumor-targeting capability.

Heparin-coated MNPs have been developed for the enhancement of cellular transduction [78]. Magnetically guided adeno-associated virus was developed to enhance gene delivery to HEK293T and PC12 cell lines. Heparin-coated MNPs were used as a moiety to immobilize the virus. With application of a magnetic field, the heparin-coated MNPs were rapidly internalized, producing efficient cellular transduction.

### 5.6. Mannan

**Figure 5:**
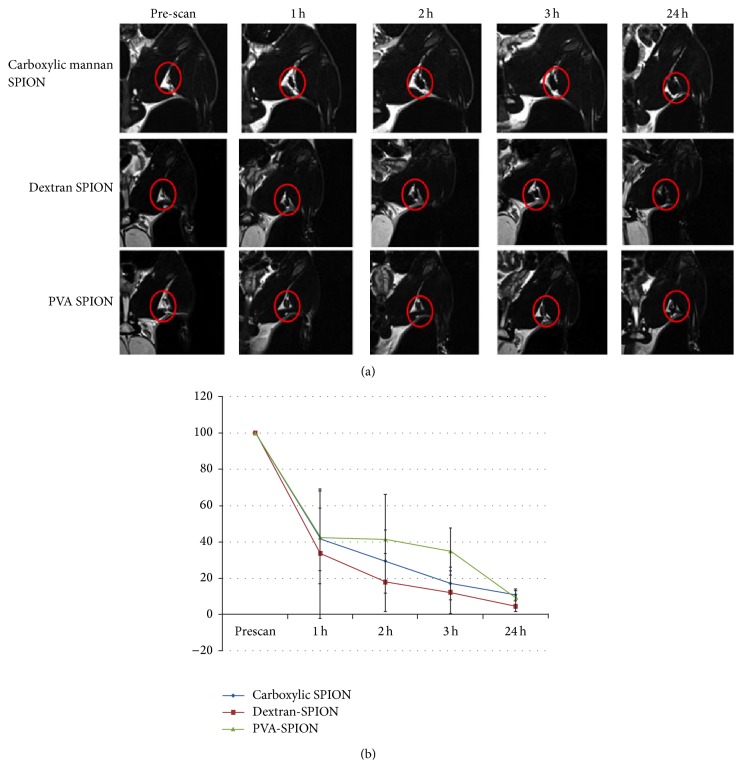
Magnetic resonance images (a) and signal intensity profile (b) of rat popliteal lymph nodes after local injection of foot pads. Reprinted with permission from [66].

Mannan is a polymer of the sugar mannose, which is of plant origin. In other words, it is a storage polysaccharide. It occurs in a variety of plants and the cell walls of some fungi. It is composed of highly branched polysaccharides with *α* (1→2) and *α* (1→3) linked mono-, di-, and trimannopyranose side chains attached to the backbone of *α* (1→6)-linked mannopyranose [108]. The chemical structure of mannan is shown in Figure 3. Apart from the other polysaccharides, it has the distinct advantage of being easily recognized by the mannose receptor, which is expressed on the surface of antigen-presenting cells; therefore, the uptake of mannose-bearing particles via mannose-mediated endocytosis and phagocytosis is facilitated in these cells. Our group constructed carboxylic mannan-coated- (CM-) SPIONs that were targeted to immune cells for the purpose of lymph-node-specific MRI [66]. These nanoparticles were fabricated to target antigen-presenting cells, including macrophages, via the specific interaction between mannose on the surface of the coated SPIONs and mannose receptors expressed on the antigen-presenting cells (APCs). The mannan was modified to carboxylic mannan by the introduction of an aldehyde group via oxidation and then the conversion of the aldehyde group to a carboxylic group. The CM-SPIONs were synthesized via the alkaline coprecipitation of FeCl_3_·6H_2_O and FeCl_2_·4H_2_O. Peritoneal macrophages isolated from Balb/c mice were used to investigate the intracellular uptake of CM-SPIONs (Figure 5). The* in vivo* uptake of CM-SPIONs was tracked by MRI after subcutaneous injection of rats. MRI analysis revealed that the injected CM-SPIONs accumulated in the popliteal lymph nodes, which was further confirmed by Prussian blue staining.

### 5.7. Pullulan

Pullulan is a water soluble, neutrally charged polysaccharide consisting of maltotriose units and is produced from starch by the fungus* Aureobasidium pullulans*. The three glucose units are connected via an *α*-1,4 glycosidic bond, while the consecutive maltotriose units are connected to each other via an *α*-1,6 glycosidic bond (Figure 3). Pullulan is widely used for liver-targeted therapies [109]. Pullulan has also been used as a carrier for oral drug delivery systems because it is not attacked by the digestive enzymes in the human gut [110]. MNPs coated with pullulan have been synthesized via the coprecipitation method, with subsequent cross linking of the polymer chain using glutaraldehyde [111]. Another method for coating MNPs with pullulan uses the amphiphilic derivative of pullulan, pullulan acetate (PA) [67]. The advantage of this method is that it does not require any cross linker, and any adverse effect of cross linker on the body can be avoided. Moreover, it is a robust and easy method with a variety of ways for controlling the size of particles.

### 5.8. Starch

Starch is an abundant biodegradable and inexpensive polysaccharide that occurs naturally as discrete particles known as granules, which most green plants store as an energy source. It consists of many glucose units joined via glycosidic bonds, as shown in Figure 3. Starch-based formulations have also been used for nasal administration [112]. MNPs coated with starch were prepared by the coprecipitation method followed by chemical cross linking of thiolated starch using glutaraldehyde [113].

## 6. Conclusions and Future Perspectives

Polymer-coated magnetic particles facilitate the delivery of therapeutic agents and have a high concentration of available functional groups that can be utilized for further bioconjugation with cell-targeting agents. The polymer coatings of magnetic particles increased their biocompatibility, stability, and concentration in the* in vivo* circulation. Moreover, for specific targeting and imaging, particles designed for magnetofection-based gene delivery have been shown to be promising candidates for dual roles as imaging contrast agents and as therapeutic agents.

Although polymer-coated magnetic particles appear to have significant potential for simultaneous imaging and therapeutic gene delivery, there are many obstacles to overcome before applying this technique to the clinic. For example, studies conducted in small animals showed greater potential for particle targeting than studies in larger animals and humans. It is much more difficult to target sites located farther from the magnetic source. Another obstacle for using polymer-coated magnetic particles for gene delivery is the inability of the MNPs to express effectively at the target site. Future research should be focused on upgrading the use of magnetic particles for other applications such as nanoarticulation and nanomodulation of the cells via cell nanoparticle interaction. While magnetic nanoparticle targeting might not be applicable to all medical conditions, with further development, MNPs should become more effective tools for treating a variety of diseases.

## Figures and Tables

**Table 1:** Properties of polysaccharides commonly used for coating or encapsulation of MNPs.

Polysaccharide	Natural source	Charge	Functional groups
Alginate	Distributed widely in the cell walls of brown algae	Negative	OH COOH

Chitosan	From the exoskeletons of shrimp and other crustaceans treated with sodium hydroxide	Positive	OH NH_2_

Dextran	First discovered by Louis Pasteur as a microbial product in wine	Neutral	OH

Hyaluronic acid	Distributed widely throughout connective, epithelial, and neural tissues	Negative	OH COOH

Heparin	Extracted from animal tissues	Negative	OH OSO_3_H

Mannan	Plant polysaccharide a form of storage polysaccharide	Neutral	OH

Pullulan	Produced from starch by the fungus *Aureobasidium pullulans *	Neutral	OH

Starch	Produced by most green plants to store energy	Neutral	OH

**Table 2:** Various strategies used for polysaccharide-coated MNPs that have been used for imaging.

Polysaccharide	Modification	*In vitro*/*in vivo *	Reference
Alginate	Alginate-poly-L-lysine-alginate (APA)	C2C12 myoblast cell line implanted into the abdominal cavity of mice	[61]

Chitosan	Chlorotoxin (CTX), Polyethylene glycol (PEG) Near-IR fluorophore (NIRF), Cy.5.5	Brain Autochthonous medulloblastomas in genetically engineered ND2: SmoA1 mice	[62]

Dextran	FITC-derivatized Tat peptide Chelator DTPA	Hematopoietic and neural progenitor cells	[63]

Hyaluronic acid	Dopamine	HCT116 NIH3T3 cells	[64]

Heparin	Gold-deposited Glycol chitosan Pluronic F-68	Tumor-bearing mice, SCC-7 (squamous cell carcinoma) cells were induced in male C3H/HeN mice by subcutaneous injection	[65]

Mannan	Carboxylic	Subcutaneous injection in a rat model	[66]

Pullulan		L929 cells KB cells with hyperthermic effect	[67]

Starch	Poly(ethylene glycol) (PEG)	Male Fisher 344 rats bearing 9L-glioma brain tumors	[68]

**Table 3:** Strategies for polysaccharide coating MNPs for gene delivery.

Polysaccharide	Modification	Gene	*In vitro*/*in vivo *	Reference
Chitosan	Polyethylenimine (PEI) Poly(ethylene glycol) (PEG)	Enhanced green fluorescent protein (EGFP) plasmid DNA	C6 cells Xenograft C6 tumors	[69]

Chitosan	Hexanoyl chloride-modified	Viral gene (Ad/LacZ)	K562 cells intestine and lung of balb-c mice	[70]

Chitosan	Poly(ethylene glycol) (PEG)	Enhanced green fluorescent protein (EGFP) plasmid DNA	Human liver carcinoma cells (HepG2),	[71]

Chitosan		MDR1 siRNA	BT325 cells	[72]

Chitosan		Enhanced green fluorescent protein (EGFP) plasmid DNA	Human embryonic kidney 293 (HEK293) cells Human A549 lung adenocarcinoma Human lung diploid cell line WI-38 (normal human fibroblast) cells Mouse heart and kidney	[73]

Dextran	Carboxymethyl	Green fluorescent protein (GFP) plasmid	Human bladder cancer BIU-87 cells	[74]

Dextran	Mouse anti-human *β*-HCG monoclonal antibody	GAPDH AS-ODN	JEG-3 JAR RL95-2 Hela HepG2 Lovo cells Nude mice	[75]

Dextran		NK4 plasmid DNA		[76]

Hyaluronic acid	Polyethylenimine (PEI)	VR1020-PyMSP119 VR1020-YFP plasmids	COS-7 cells C57BL/6 mice	[77]

Heparin	Wild-type AAV2 AAVr3.45	Green fluorescent protein (GFP)	HEK293T PC12	[78]
